# Cost-Effectiveness Analysis of Pan-Genotypic Sofosbuvir-Based Regimens for Treatment of Chronic Hepatitis C Genotype 1 Infection in China

**DOI:** 10.3389/fpubh.2021.779215

**Published:** 2021-12-09

**Authors:** Hui Jun Zhou, Jing Cao, Hui Shi, Nasheen Naidoo, Sherehe Semba, Pei Wang, Yi Fan Fan, Shui Cheng Zhu

**Affiliations:** ^1^Department of Public Administration, Business School, University of Shanghai for Science and Technology, Shanghai, China; ^2^Department of Pathology, Stellenbosch University, Cape Town, South Africa; ^3^Faculty of Science, Dar es Salaam University College of Education, University of Dar es Salaam, Dar es Salaam, Tanzania; ^4^School of Public Health, Fudan University, Shanghai, China; ^5^Key Lab of Health Technology Assessment, National Health Commission of China (Fudan University), Shanghai, China

**Keywords:** hepatitis C-chronic, cost-utility analysis, sofosbuvir/velpatasvir, sofosbuvir/ledipasvir, economic modeling, incremental cost-effectiveness ratio, net monetary benefit

## Abstract

**Background:** Hepatitis C virus (HCV) genotype 1 is the most prevalent HCV infection in China. Sofosbuvir-based direct antiviral agent (DAA) regimens are the current mainstays of treatment. Sofosbuvir/velpatasvir (SOF/VEL) and sofosbuvir/ledipasvir (SOF/LDV) regimens became reimbursable in China in 2020. Thus, this study aimed to identify the optimal SOF-based regimen and to inform efficient use of healthcare resources by optimizing DAA use in treating HCV genotype 1.

**Methods and Models:** A modeling-based cost-utility analysis was conducted from the payer's perspective targeting adult Chinese patients with chronic HCV genotype 1 infection. Direct medical costs and health utilities were inputted into a Markov model to simulate lifetime experiences of chronically infected HCV patients after receiving SOF/LDV, SOF/VEL or the traditional strategy of pegylated interferon (pegIFN) + ribavirin (RBV). Discounted lifetime cost and quality adjusted life years (QALYs) were computed and compared to generate the incremental cost utility ratio (ICUR). An ICUR below the threshold of 31,500 $/QALY suggests cost-effectiveness. Deterministic and probabilistic sensitivity analyses were performed to examine the robustness of model findings.

**Results:** Both SOF/LDV and SOF/VEL regimens were dominant to the pegIFN + RBV regimen by creating more QALYs and incurring less cost. SOF/LDV produced 0.542 more QALYs but cost $10,390 less than pegIFN + RBV. Relative to SOF/LDV, SOF/VEL had an ICUR of 168,239 $/QALY which did not meet the cost-effectiveness standard. Therefore SOF/LDV was the optimal strategy. These findings were robust to linear and random variations of model parameters. However, reducing the SOF/VEL price by 40% would make this regimen the most cost-effective option.

**Conclusions:** SOF/LDV was found to be the most cost-effective treatment, and SOF/VEL was also economically dominant to pegIFN + RBV. These findings indicated that replacing pegIFN + RBV with DAA regimens could be a promising strategy.

## Introduction

Hepatitis C virus (HCV) infection is an escalating global health concern. China alone has contributed 10 million cases of chronic HCV (CHC) infection accounting for 7% of global infections (1). HCV genotype 1 is the most prevalent HCV infection in China (2–4). Within the current healthcare system, HCV epidemic and resulting complications such as cirrhosis, decompensated cirrhosis (DCC), hepatocellular carcinoma (HCC) and liver transplantation (LT) are expected to increase in the next 10 years (5). This presents a significant public health challenge to the Chinese government and subsequently to the WHO goal of global HCV elimination by 2030 (6).

Similar to other developed countries, China has also entered the era of direct acting antiviral agents (DAAs) for HCV treatment. DAAs are highly effective in treating HCV as sustained virologic response (SVR) rates of these drugs are generally higher than 95% (7). Currently most DAAs have been approved and are marketed in China (8). DAAs are now becoming the first-line treatment option for HCV infection. Sofosbuvir (SOF)-based regimens appear to be the most widely used treatment strategy largely because sofosbuvir/velpatasvir (SOF/VEL) was the first and only DAA included in the National Essential Drug List (October 2018) in China (9). In 2020, SOF/VEL and sofosbuvir/ledipasvir (SOF/LDV) inclusion were negotiated by the government and their prices were cut dramatically (10). Concurrently SOF/VEL and SOF/LDV were enrolled in the National Reimbursement Drug List (NRDL) where a range of 70–90% of the cost was entitled for government reimbursement (11).

This action marked the commitment of the Chinese government to fight the HCV epidemic. It is likely that the SOF/LDV and SOF/VEL regimens will become the mainstays of CHC treatment in the coming years. Clinical guidelines to date do not mention which SOF-based regimen is the preferred strategy, but instead recommend them equally as the pan-genotypic DAA for treatment of CHC (12). However, SVR rates are comparable between SOF/LDV and SOF/VEL treatment strategies (13, 14). Therefore, clinicians would face difficulties in choosing the best regimen if their decision were based solely on the efficacy or effectiveness. It is valuable for clinicians to understand the economic value of different treatments as another facet toward improving clinical decision-making. In addition, clinical decisions based on the economic evidence would enhance efficiency in allocating healthcare budgets.

Economic evaluations of SOF/LDV and SOF/VEL have been performed previously in patients with HCV genotype 1 infection. Compared to IFN-based regimens, a 12-week course of SOF/LDV was cost-saving for patients with cirrhosis (15), but was not cost-effective in treatment naïve patients (16). In a cost-utility analysis comparing SOF/VEL with other DAA regimens (17), SOF/VEL was not the most cost-effective option for patients with HCV genotype 1b and was less advantageous compared to elbasvir/grazoprevir and paritaprevir/ritonavir + dasabuvir regimens. However, these studies were conducted before the price negotiation, and therefore the drug prices used were not the most up-to-date. In a recent modeling-based economic analysis after the new price policy was implemented, SOF/LDV, SOF/VEL and other two DAA regimens were compared (57). Although the lifetime cost was the lowest for the SOF/LDV regimen, the SOF/VEL regimen was the most cost-effective strategy for treating HCV genotype 1 infection. However, this study did not include an IFN-based regimen as the reference case despite the regimen still in use in many medical institutes. More studies are necessary to build a strong foundation of economic evidence to assist with clinical decision making on optimum CHC treatment.

With the two drugs included in the NRDL, it is necessary to re-evaluate the comparative advantages of SOF/VEL and SOF/LDV regimens in relation to the traditional standard of care (SoC). We designed this cost-utility analysis based on a Markov model to evaluate and compare SOF/LDV, SOF/VEL and SoC regimens in terms of costs and quality-adjusted life years (QALYs). We aimed to (1) identify the optimal SOF-based regimen to aid clinicians in making informed clinical decisions; (2) To inform the efficient use of limited healthcare resources by optimizing DAA use in clinical practice.

## Materials and Methods

### Candidate Strategies and Primary Clinical Outcome

Similar to the previous studies (18–20), the SoC in China consisting of pegIFN + RBV for 48 weeks was set as the reference case. Although the decade-long pegIFN-based regimens are now less popular, they are still in use in many areas of China. Candidate strategies were SOP/LDV (400 mg/90 mg) once daily for 12 weeks, as per the Chinese Clinical Guideline for Treatment and Prevention of Hepatitis C Infection (12). The SOF/VEL regimen prescribes SOF/VEL (400/100 mg) for 12 weeks as a once-daily oral administration. Another DAA, elbasvir/grazoprevir, which was also enrolled in NRDL in 2020 was not selected for comparison mainly because it is not pan-genotypic.

The primary clinical endpoint was the SVR rate at 24 weeks (SVR-24) after a course of drug treatment. If SVR-24 was not reported, SVR-12 was taken as a substitute because of the high consistency between SVR-12 and SVR-24 (15, 21, 22). SVR refers to undetectable HCV RNA in blood indicating that patients have cleared the virus. Data of SVR were all extracted from clinical trial or real-world studies on Chinese populations (Table 1). A serious adverse event (SAE) refers to any unexpected clinical event occurring during drug treatment that was reported as a SAE in the original study. Failing to explicitly model SAEs would underestimate the value of the DAA regimens as they are associated with a lower SAE rate. SAE rates were inputted in the model and extracted from the same studies of SVR to maintain consistency between model parameters.

**Table 1:** Clinical profile of candidate strategies.

	**Point estimate**	**Lower limit**	**Higher limit**	**Distribution**	**Distribution parameter**		**References**
**SVR rate (%)**							
PegIFN + RBV	57.88	49.48	67.29	beta	169	123	(23)
SOF/LDV	99.61	99.02	100	beta	996	4	(15, 21, 22, 24)
SOF/VEL	100	99.46	100	beta	998	2	(21, 25)
**SAE rate (%)**							
PegIFN + RBV	8.7	2.37	22.26	beta	4	42	(25)
SOF/LDV	2.08	0.90	4.06	beta	8	376	
SOF/VEL	0.80	0.17	2.32	beta	3	372	

*PegIFN, pegylated interferon; RBV, ribavirin; SAE, serious adverse event; SOF/LDV, sofosbuvir/ledipasvir; SOF/VEL, sofosbuvir/velpatasvir; SVR, sustained virologic response*.

Given that higher SVR is the main advantage distinguishing DAA regimens from IFN-based regimens, our model made efforts to fully capture multiple effects associated with SVR, including clinical and cost aspects. The clinical effect of SVR was represented with fibrosis regression (F4–F3, F3–F2), and reduced risk of HCC, DCC and liver-related death (LD) (26–28) (Table 2). In addition, the cost of managing each stage of fibrosis was reduced compared to patients without SVR (33). However, once patients developed DCC, HCC or required a LT, we assumed that the past SVR would not have an effect on subsequent disease progression.

**Table 2:** Inputted model parameters for costs, transition probability, utility, and comprehensive effect of sustained virologic response.

**Input parameters**	**Point estimate**	**Lower limit**	**Higher limit**	**Distribution**	**Distribution parameter^a^**		**References**
**Price of drugs ($)**							
SOF/LDV (400/90 mg)	10.86	5.5	16.5	–	–	–	(8, 29)
SOF/VEL (400/100 mg)	22.04	11	33	–	–	–	
PegIFN (50 ug)	0.24	0.04	1.95	–	–	–	
RBV (100 mg)	35.95	16.56	97.33	–	–	–	
**Direct medical cost of HCV treatment ($)**							
F0-3	1,971	593	7,565	Gamma	3,344	1,162	(16, 18, 19, 30, 31)
Cirrhosis	4,760	884	26,766	Gamma	6,182	4,314	
HCC	28,936	4,618	97,943	Gamma	28,936	15,554	
LT during 1st year	147,854	36,765	76,923	Gamma	147,854	64,591	(18, 31, 32)
LT 2nd year onwards	24,374	7,352	9,457	Gamma	24,374	10,560	(18, 33)
DCC	14,477	2,589	50,441	Gamma	14,477	7,975	(18, 19, 30)
**Transition probability (%)**							
F0–F1	10.70	9.70	11.80	Beta	1,070	8,930	(34)
F1–F2	8.20	7.40	9.10	Beta	82	918	
F2–F3	11.70	10.70	12.90	Beta	117	883	
F3–Cirrhosis	11.60	10.40	13.10	Beta	116	884	
Cirrhosis to DCC	4.28	3.80	5.30	Beta	43	957	(19, 30, 35)
Cirrhosis to HCC	1.90	1.70	2.10	Beta	19	981	(30, 36)
DCC to LT	3.76	0.03	10.40	Beta	3.76	96.24	(36)
DCC to death during 1st year diagnosis	6.90	2.60	12.90	Beta	14.7	85.3	
DCC to HCC	3.75	2.10	6.80	Beta	37.5	962.5	
HCC to LT	2.12	0.05	4.00	Beta	21.2	978.8	(18, 37)
HCC to death	44.80	34.90	57.60	Beta	49.6	110.4	
Death rate of year 1 post-LT	23.51	20.90	26.25	Beta	235.1	764.9	(37)
Death rate of year 2 post-LT	8.22	6.58	10.08	Beta	82.2	917.8	
Death rate of year 3 post-LT	8.22	6.58	10.08	Beta	82.2	917.8	
All-cause death	Age-gender specific mortality of China life table of 2019						(38)
**Utility**							
F0/F1 without SVR	0.878	0.751	0.985	Normal	0.878	0.039	(16, 18, 39)
F2/F3 without SVR	0.863	0.701	0.985	Normal	0.863	0.0473	
Cirrhosis	0.792	0.67	0.907	Normal	0.792	0.0395	
DCC	0.576	0.41	0.66	Normal	0.576	0.0417	
HCC	0.685	0.532	0.821	Normal	0.685	0.0482	
LT during 1st year	0.663	0.563	0.8	Normal	0.663	0.0395	
LT from 2nd year onwards	0.773	0.636	0.85	Normal	0.773	0.0357	
F0/F1 with SVR	0.928	0.806	1	Normal	0.928	0.0323	
F2 with SVR	0.911	0.791	1	Normal	0.911	0.0348	
F3 with SVR	0.893	0.766	1	Normal	0.893	0.0390	
Cirrhosis with SVR	0.85	0.722	0.955	Normal	0.85	0.0388	
**Comprehensive effect of SVR**							
Cost reduction (RR)	0.709	0.592	0.855	log Normal	−0.344	0.094	(33)
Fibrosis regression (%)	13.66	8.39	20.17	Beta	137	863	(40)
**Reduced transition from cirrhosis to end stage liver disease (Hazard Ratio)**							
Cirrhosis to DCC	0.16	0.04	0.59	log Normal	−1.833	0.687	(26–28, 41)
Cirrhosis to HCC	0.24	0.18	0.31	log Normal	−1.561	0.134	
Cirrhosis to death	0.23	0.1	0.52	log Normal	−1.470	0.421	
**Other parameters**							
Discount rate (%)	3	0	5				(42)
WTP ($/QALY)	31,500			–			(43)

a *The parameters of gamma and beta distributions are alpha and beta; The parameters of normal distributions are mean and standard error; The parameters of lognormal distributions are logarithmic mean and standard error. DCC, decompensated cirrhosis; METAVIR fibrosis score; F0, CHC with no fibrosis; F1, portal fibrosis without septa; F2, portal fibrosis with few septa; F3, numerous septa without cirrhosis; HCC, hepatocellular carcinoma; HCV, hepatitis C virus; LT, liver transplant; pegIFN, pegylated interferon; RBV, ribavirin; SAE, serious adverse event; SOF/LDV, sofosbuvir/ledipasvir; SOF/VEL, sofosbuvir/velpatasvir; SVR, sustained virologic response; QALY, quality adjusted life year; WTP, Willingness-to-pay*.

### Study Population and Its Epidemic Features

As HCV genotype 1 is the most prevalent infection in China (3), the target population was identified as adult CHC patients with HCV genotype 1 infection who were naïve to drug treatment. The mean age was 45 years old as informed by the study investigating current CHC patients under drug treatment (4, 44). Pan-genotypic DAAs are normally recommended to a wide range of patients.

### Model Construction

**Figure 1:**
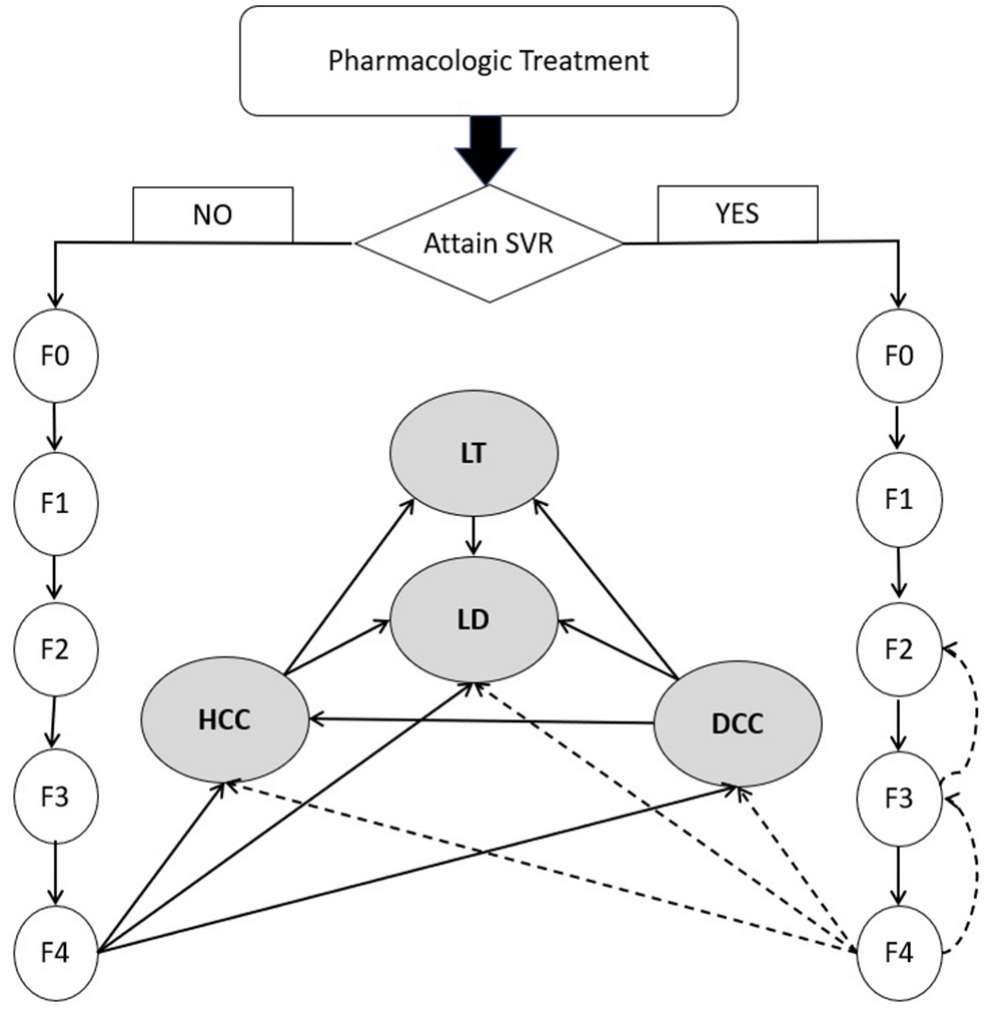
Natural history of chronic hepatitis C virus infection. DCC, decompensated cirrhosis; F0, CHC with no fibrosis; F1, portal fibrosis without septa; F2, portal fibrosis with few septa; F3, numerous septa without cirrhosis; F4, cirrhosis; HCC, hepatocellular carcinoma; HCV, hepatitis C virus; LD, liver-related death; LT, liver transplant; SVR, sustained virologic response. Dashed line represents the slow transition or regression due to SVR.

Conceptualized on the life journey of a CHC patient (Figure 1), a decision-analytic model was constructed to compare three treatment strategies in terms of cost and effectiveness. As data about productivity loss or out-of-pocket spending were rarely reported in a Chinese setting, we adopted a payers' perspective instead of a wider societal perspective focusing on health outcomes experienced by the target population only and the cost for medical services provided to them (45). Markov models were specifically developed for each regimen. After a complete course of drug treatment, a patient may or may not achieve SVR, but the disease would continue to advance. The initial pathological change is the fibrosis caused by chronic HCV-induced inflammation. After years or decades of fibrosis, cirrhosis occurs and further develops into end-stage liver diseases like DCC, HCC or LT. Patients with end-stage liver disease are at higher risk for all-cause mortality than the general population, in addition to excessive mortality due to liver-related complications. Markov models simulated the lifetime experience of the target population since the drugs were administrated. Eleven Markov states (health states) were defined, i.e., METAVIR fibrosis scores F0 (CHC patients without fibrosis), F1 (portal fibrosis without septa), F2 (portal fibrosis with few septa), F3 (numerous septa without cirrhosis) and F4 (cirrhosis), DCC, HCC, LT, LD and all-cause death. Patients who achieved SVR and those without SVR shared the same clinical journey but experienced different risks of downstream clinical outcomes. The Markov cycle length was set as 1 year with half-cycle correction applied to adjust for the overestimation of life expectancy. The model adopted a lifetime horizon where 99% of the target population died of either LD or other causes. The models were built using Treeage Pro Suite 2021 (TreeAge Pro 2021, R1. TreeAge Software, Williamstown, MA; software available at http://www.treeage.com).

The Markov models made the following assumptions:

(1) Fibrosis progressed in a linear path from no fibrosis to cirrhosis. The jumping among fibrosis stages was not considered (46).
(2) The risk of HCC was considered negligible in pre-cirrhosis stages of liver disease.
(3) Fibrosis would progress irrespective of past SVR status. Patients who achieved SVR would experience slower progress than those not achieving SVR (40).
(4) SVR represented the overall clinical efficacy of therapeutic regimens. Virologic breakthrough, relapse or drug discontinuation during treatment were not explicitly modeled.
(5) Long-term health outcomes were associated with SVR status alone regardless of the drugs received during past treatment.
(6) Reinfection or retreatment was considered of minor importance and was not modeled explicitly (47).

### Data Collection and Synthesis

Data were retrieved mainly from literature. Meta-analytic techniques, such as inverse variance weighting, were used to generate model parameters for which multiple estimates were available. Data sources were judged in terms of relevance, internal validity and transferability to the Chinese healthcare setting. To improve the relevance of model outputs to the Chinese healthcare setting, efforts were made to retrieve data on Chinese populations.

#### Transitions Among Health States

Fibrosis progression was estimated from an up-to-date meta-analysis of the natural history of CHC (34). For patients who achieved SVR after pharmacologic treatment, fibrosis regression was incorporated into their pathway as studies have reported SVR-related regression from cirrhosis to fibrosis, and from F3 back to F2 (40). However, once patients developed DCC, HCC or received a LT, we assumed that SVR would not be relevant to the patient's further prognosis. Data about disease progression after cirrhosis were synthesized from multiple studies (26–28, 41). The probability of requiring a LT for patients with DCC or HCC was extracted from the data of the China Liver Transplant Registry (37) (Table 2).

#### Cost and Utility

In line with the payers' perspective, only direct medical costs were considered which included the cost for drug treatment, disease monitoring, HCV testing, hospitalization, LT and consumption of other healthcare services as part of CHC management. Drug prices displayed on the official government procurement website (8) were used because these prices were negotiated between health authorities and pharmaceutical companies and represented the acquisition costs of public medical institutions. We took the average of province-specific prices to represent the national level. Costs for health states were retrieved from studies on Chinese patients. Costs reported in different years were adjusted by the annual CPI to the 2020 constant US dollar ($).

Health utility was represented by the health-related quality of life (QoL) scores in EQ-5D (Table 2). Data were extracted from the literature and adjusted to reflect the utility of Chinese patients in different health states (18, 39).

### Model Analysis

#### Base-Case Analysis

Base-case analysis was conducted where the best estimates were assumed for all parameters in the model. Cost was assigned for each Markov state to reflect healthcare resource consumption in managing the specific HCV stages. A QoL score was assigned to reflect patients' utility in that health state. As the Markov models simulated the life experience of the cohort, the cost and utility of Markov states accumulated over time. Total cost and quality adjusted life years (QALYs) were computed for the three strategies, SOF/LDV, SOF/VEL and pegIFN + RBV. The comparative cost-effectiveness was gauged as the incremental cost-utility ratio (ICUR). The cost difference between the three regimens was divided by the inter-regimen difference in QALYs. The ICUR represents the amount that a jurisdiction needs to pay for one additional QALY gained. In the modeling, both cost and utility have been discounted at an annual rate of 3% to the constant dollar in 2020 (42). Following the WHO rule, the willingness-to-pay (WTP) threshold was set as three per capita GDPs, i.e., $31,500 in 2020, which is the benchmark for cost-effectiveness informing the maximum amount a country is willing to pay to generate one QALY. The regimen with an ICUR < $31,500 relative to the last optimal regimen was considered cost-effective. Net monetary benefit (NMB) was also calculated for each strategy by deducting cost from the product of the QALYs and WTP threshold. NMB represents the health benefit created by each regimen at the given WTP standard. The regimen creating the most NMB was the most cost-effective strategy.

#### Sensitivity Analysis

**Figure 2:**
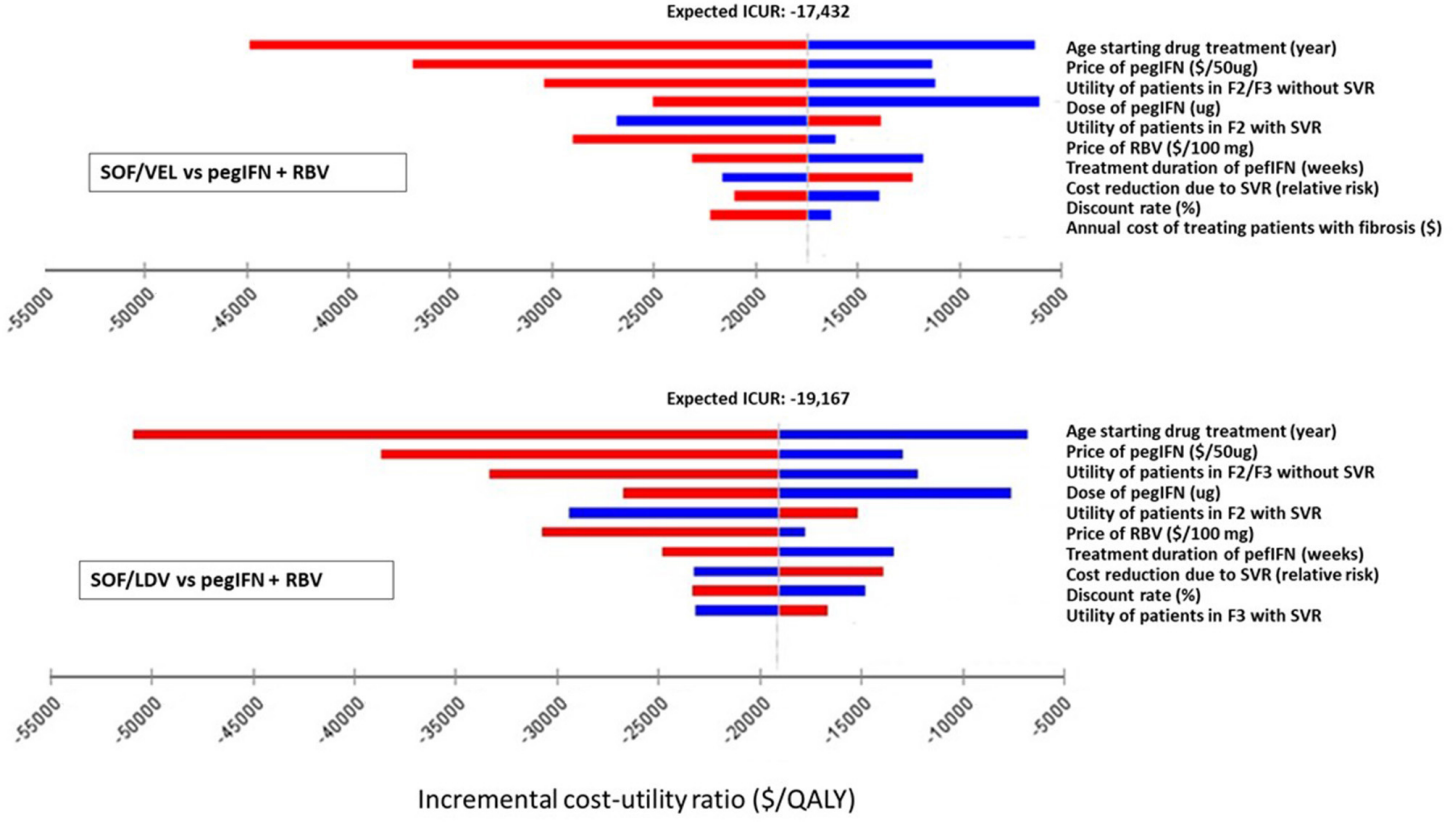
Impact of the 10 most influential parameters on cost-effectiveness of SOF/LDV and SOF/VEL vs. pegIFN + RBV. F2, portal fibrosis with few septa; F3, numerous septa without cirrhosis; ICUR, incremental cost utility ratio; PegIFN, pegylated interferon; QALY, quality adjusted life years; RBV, ribavirin; SOF/LDV, sofosbuvir/ledipasvir; SOF/VEL, sofosbuvir/velpatasvir; SVR, sustained virologic response.

**Figure 3:**
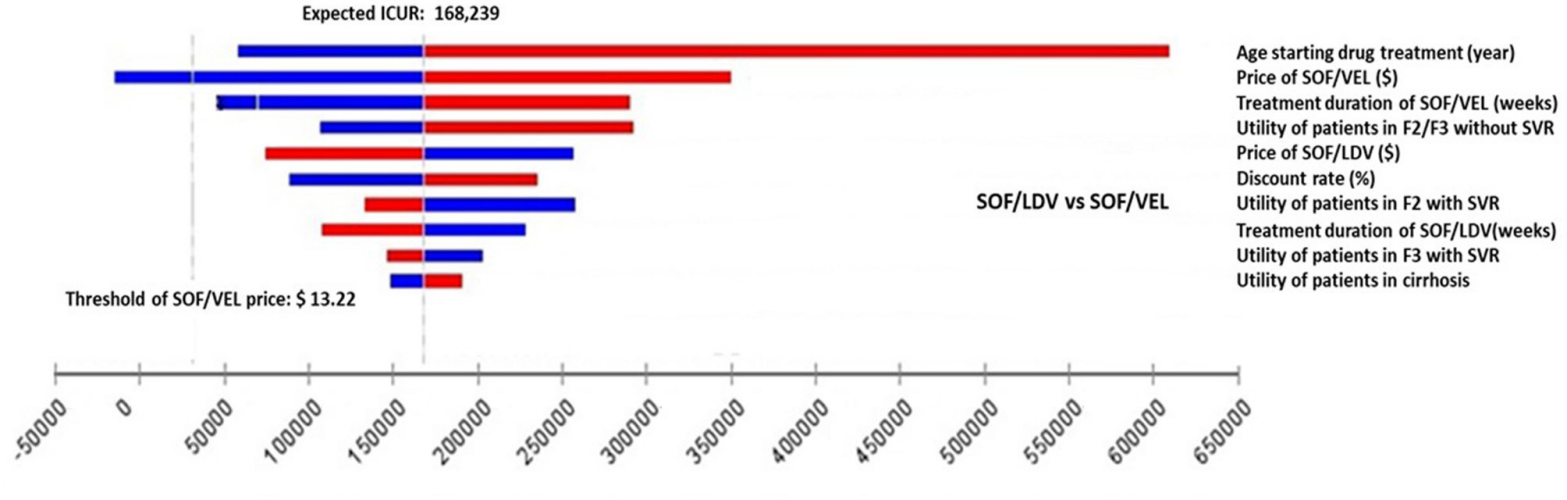
Impact of the 10 most influential parameters on cost-effectiveness of SOF/VEL vs. SOF/LDV. F2, portal fibrosis with few septa; F3, numerous septa without cirrhosis; ICUR, incremental cost utility ratio; QALY, quality adjusted life years; SOF/LDV, sofosbuvir/ledipasvir; SOF/VEL, sofosbuvir/velpatasvir; SVR, sustained virologic response.

One-way deterministic sensitivity analysis (DSA) was performed on individual parameters to examine how their variations were capable of changing the results from the base-case analysis. The model used serial values within the range of each parameter one at a time and produced a set of ICURs or NMB for that parameter. The variation of ICURs or NMB represented how influential a specific parameter was to the model output. Although ranges of parameters were clinically plausible, some values were mathematically extreme so as to produce unstable and extreme ICURs. For instance, the utility of patients in F0/F1 and SVR of SOF/VEL and SOF/LDV regimens. These parameters were excluded from one-way DSA. DSA results were presented in tornado diagrams (Figures 2, 3).

Sampling uncertainty of parameters would cause some probability of incorrect model decision. This effect was evaluated using probabilistic sensitivity analysis (PSA). PSA conducted random sampling 10,000 times simultaneously on predefined distributions of each parameter to capture the total random uncertainty of model outcomes. At every sampling, cost and QALYs were compared between the three regimens and ICURs were calculated to identify the optimal regimen. With 10,000 comparisons, the probability of being cost-effective was computed for each regimen not only at the chosen WTP of $31,500/QALY, but also across a wide range of WTPs. The results of PSA were presented in the cost-effectiveness acceptability curve (CEAC) and incremental cost-effectiveness (ICE) scatter plots.

## Results

### Base-Case Analysis

As projected by our model (Table 3), pegIFN + RBV was dominated by both SOF/LDV and SOF/VEL regimens by creating more QALYs and incurring less cost. The SOF/LDV regimen appeared to be the most economical strategy for our target population. Compared to the reference case of pegIFN + RBV, SOF/LDV produced 0.542 more QALYs but spent $10,390 less on average over the lifetime of a CHC patient with HCV genotype 1. Although SOF/VEL created 0.005 more QALYs than SOF/LDV, it incurred an additional cost of $852, resulting in an ICUR of 168,239 $/QALY, which was higher than the WTP threshold of 31,500 $/QALY.

**Table 3:** Cost and effectiveness of the pegIFN+RBV, SOF/LDV, and SOF/VEL regimens in increasing order of effectiveness.

**Regimens**	**Cost ($)**	**Incremental cost ($)**	**Utility (QALY)**	**Incremental utility (QALY)**	**ICUR ($/QALY)**	**NMB ($)**
PegIFN/RBV	41,084	-	18.219	-	Reference case	560,273
SOF/LDV	30,694	−10,390	18.761	0.542	Dominant	559,580
SOF/VEL	31,547	852	18.766	0.005	168,239	532,808

*ICUR, incremental cost utility ratio; NMB, net monetary benefit; PegIFN, pegylated interferon; QALY, quality adjusted life years; RBV, ribavirin; SOF/LDV, sofosbuvir/ledipasvir; SOF/VEL, sofosbuvir/velpatasvir; SVR, sustained virologic response*.

### Deterministic Sensitivity Analysis

Within clinically and economically plausible ranges of each parameter, SOF/LDV maintained an economic advantage over pegIFN + RBV and SOF/VEL by creating the most NMB (Supplementary Table 1). In pairwise comparisons of SOF/VEL vs. pegIFN + RBV and SOF/LDV vs. pegIFN + RBV, the 5 most influential parameters were the same as shown in Figures 2, 3, i.e. age starting drug treatment, price of pegIFN, the utility of patients with fibrosis score F2 or F3, the dose of IFN, the utility of patients in F2 stage achieving SVR (Figure 2). Furthermore, SOF/VEL and SOF/LDV retained their dominance over pegIFN + RBV regardless of parameter variations as indicated by negative ICURs where pegIFN + RBV was associated with a higher cost and fewer QALYs.

However, for the comparison between SOF/VEL and SOF/LDV, the 5 most influential parameters were: the age starting drug treatment, the price of SOF/VEL, the duration of SOF/VEL treatment, the utility of patients in F2 or F3 stage and price of SOF/LDV (Figure 3). A threshold was discovered with the price of SOF/VEL. If the price of SOF/VEL decreased by 40%, then SOF/VEL would be the cost-effective alternative rather than SOF/LDV.

### Probabilistic Sensitivity Analysis

**Figure 4:**
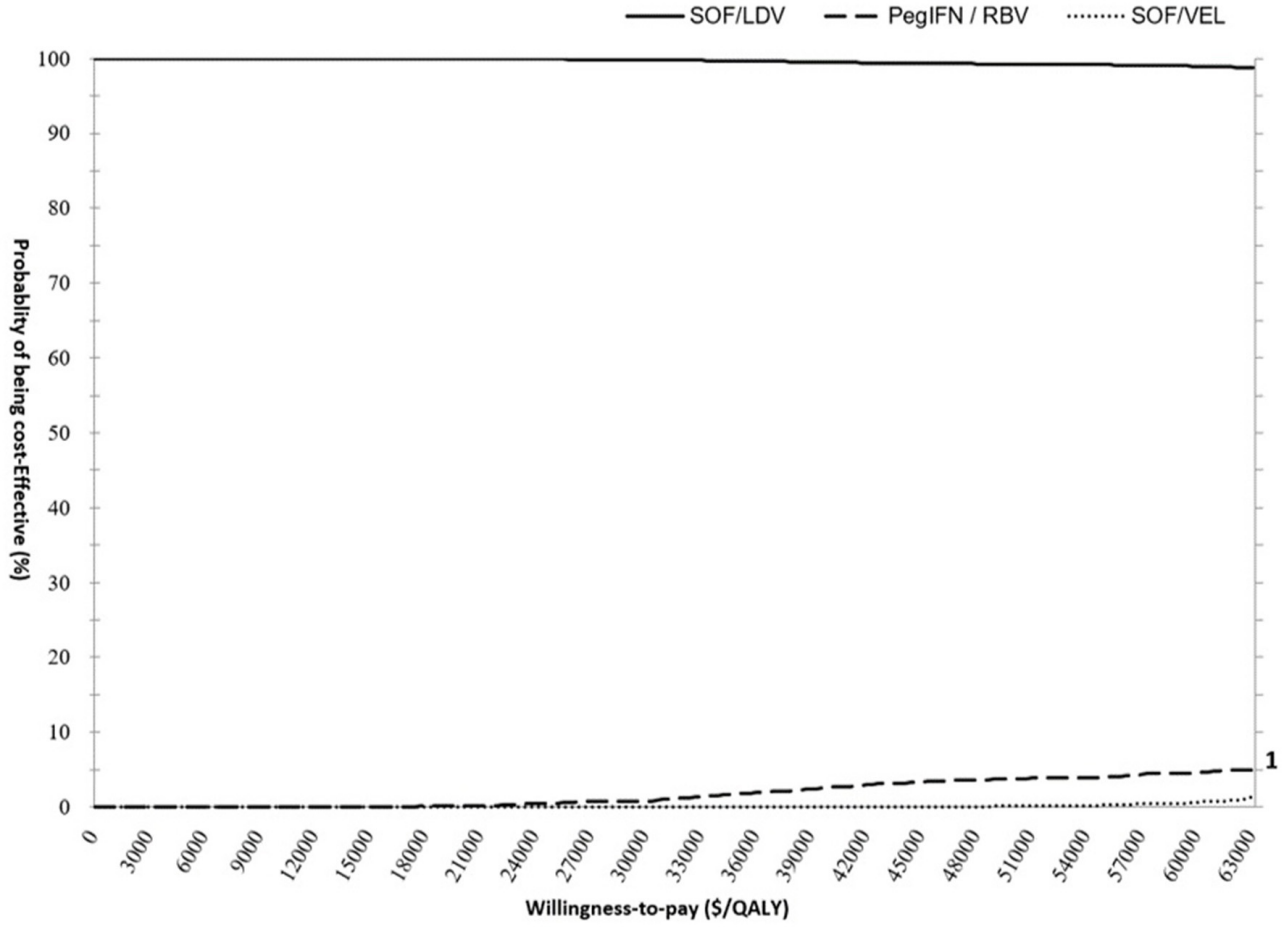
Cost- effectiveness acceptability curve (CEAC) presenting the probability of cost-effectiveness for pegIFN+RBV, SOF/LDV and SOF/VEL regimens. The scale for primary and secondary Y axis is percentage. The curves of PegIFN and SOF/VEL follow the scale of the secondary Y Axis to improve visibility. PegIFN, pegylated interferon; QALY, quality adjusted life years; RBV, ribavirin; SOF/LDV, sofosbuvir/ledipasvir; SOF/VEL, sofosbuvir/velpatasvir.

The PSA results supported the findings from the base-case analysis. As demonstrated in the CEAC (Figure 4), the acceptability curve representing SOF/LDV ran high above the curves for SOF/VEL and pegIFN + RBV highlighting that SOF/LDV dominated both pegIFN + RBV and SOF/VEL after accounting for random uncertainty associated with all parameters. At the null WTP, the probability of cost-effectiveness for SOF/LDV was 99.98%, and dropped to 99.79% at the threshold of 31,500 $/QALY.

**Figure 5:**
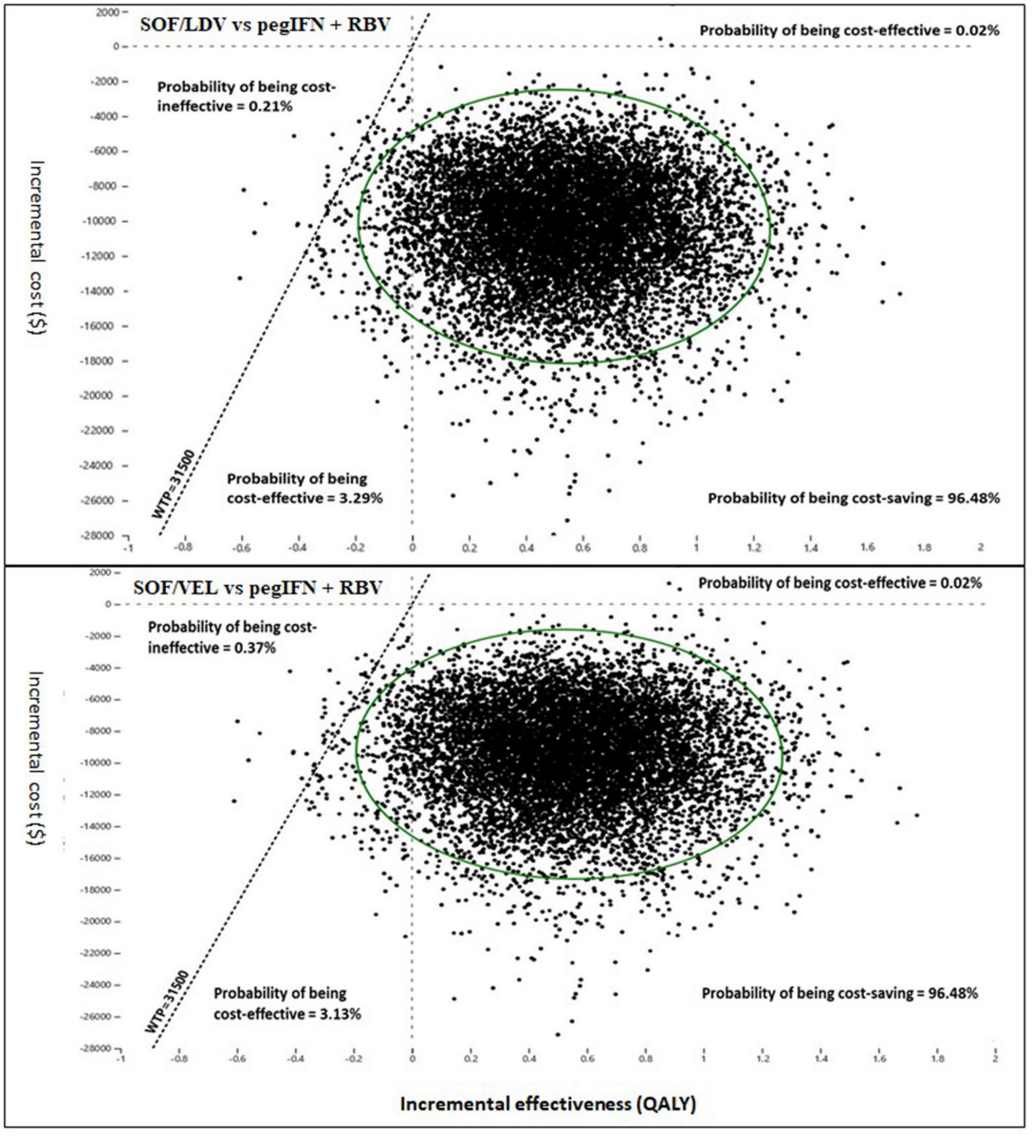
Incremental cost and effectiveness of SOF/LDV and SOF/VEL relative to pegIFN + RBV in Monte Carlo simulation (*n* = 10,000). PegIFN, pegylated interferon; QALY, quality adjusted life years; RBV, ribavirin; SOF/LDV, sofosbuvir/ledipasvir; SOF/VEL, sofosbuvir/velpatasvir; WTP, willingness-to-pay.

**Figure 6:**
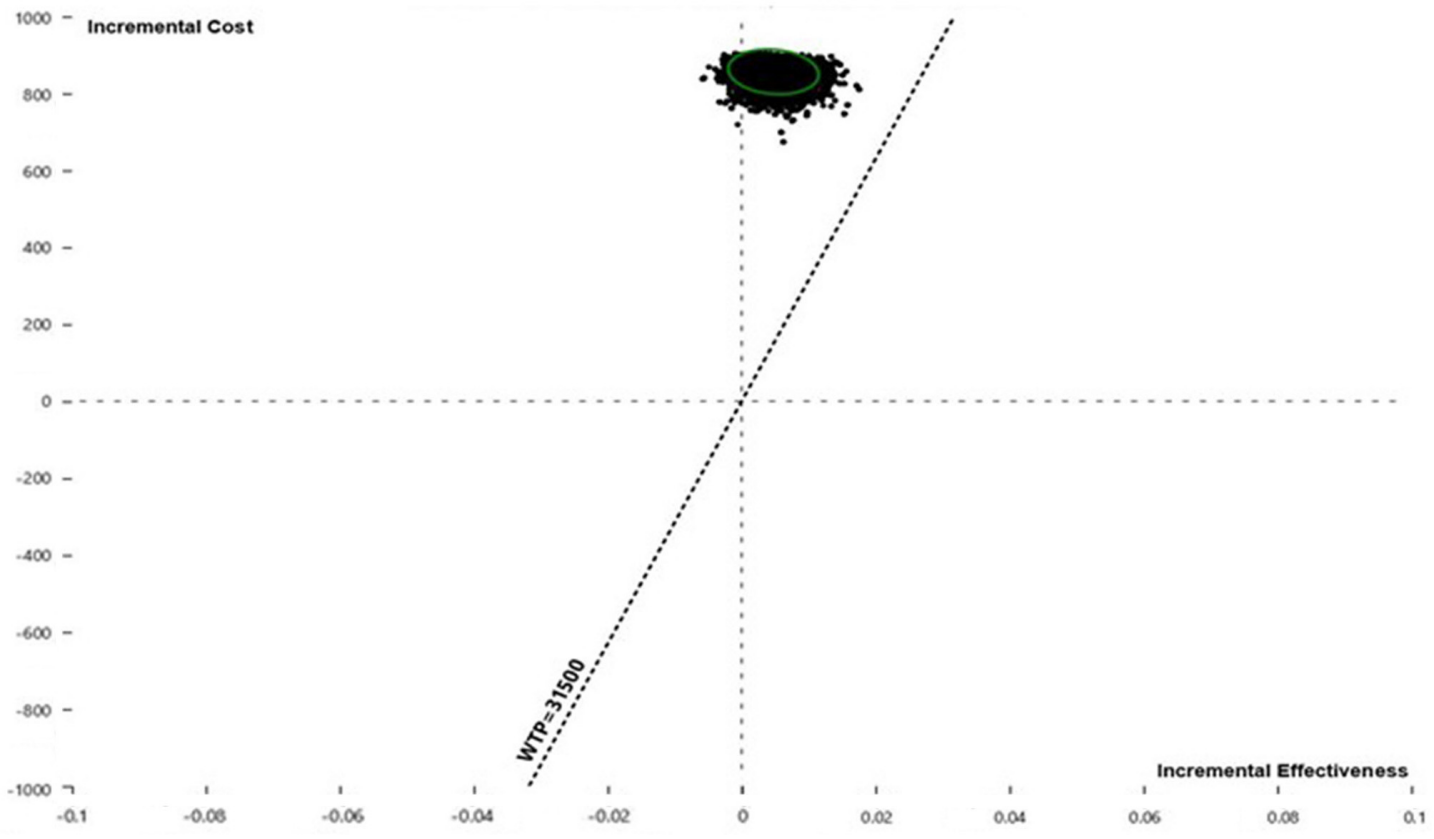
Incremental cost and effectiveness of SOF/VEL relative to SOF/LDV in Monte Carlo simulation (*n* = 10,000). QALY, quality adjusted life year; SOF/LDV, sofosbuvir/ledipasvir; SOF/VEL, sofosbuvir/velpatasvir; WTP, willingness-to-pay.

Pairwise comparisons displayed as ICE plots (Figure 5) showed that SOF/VEL and SOF/LDV obtained a similar probability composition when both were compared to pegIFN + RBV. The 95% CI ellipses fell to the right of the WTP line without any intersection, proving that SOF/LDV and SOF/VEL were statistically significantly more cost-effective than pegIFN + RBV at the current WTP standard. Relative to pegIFN + RBV, the probabilities of being cost-saving for SOF/VEL and SOF/LDV were both 96.48%. The difference between the two regimens was the probability of being cost-ineffective which were 0.37 and 0.21% for SOF/VEL and SOF/LDV respectively. The head-to-head comparison of SOF/VEL vs. SOF/LDV illustrated that SOF/LDV was either more cost-effective (96.35%) or cost-saving (3.5%). The economic advantage of SOF/LDV reached statistical significance at the current WTP as shown by the 95% ellipses (Figure 6).

## Discussion

With the inclusion of the SOF/VEL and SOF/LDV regimens in the NRDL, the use of SOF-based regimens is likely to further expand in clinical practice. To aid clinicians in their choice of the optimal regimen, this study compared SOF/LDV, SOF/VEL and pegIFN + RBV regimens for Chinese patients chronically infected with HCV genotype 1 in terms of the lifetime cost-effectiveness. Our models showed that SOF/LDV was the most cost-effective regimen among the three candidate regimens. Relative to pegIFN + RBV, SOF/LDV was cost-saving while it was cost-effective compared to SOF/VEL. Although SOF/VEL was not as cost-effective as SOF/LDV, it dominated the pegIFN + RBV regimen by spending less and generating more QALYs. Both DSA and PSA results have strengthened our primary findings. In NMB-based DSA, SOF/LDV continued to be the strategy generating the most net benefit despite the wide ICUR variations caused by parameter uncertainties (Supplementary Table 1). PSA revealed that SOF/LDV is most likely the cost-effective or cost-saving strategy. Its economic advantage was statistically significant relative to SOF/VEL and pegIFN + RBV at the current WTP standard.

It is a common belief that DAA regimens for CHC management are clinically equivalent. In our study we found that the SVRs of SOF/VEL and SOF/LDV were close enough to have overlapping 95% CI and were almost indistinguishable (Table 1). This ha1s presented a challenge to clinicians when choosing the optimal regimen. Now there is health economic evidence to assist in such clinical decision-making and further to improve resource allocation in HCV care. Our study has advanced clinical insights suggesting that SOF/LDV is preferred to SOF/VEL for the long-term management of Chinese CHC patients with HCV genotype 1 infection in the current price structure.

Our finding that the SOF/LDV regimen incurred the least lifetime cost is consistent with another study that performed head-to-head comparisons of SOF/LDV vs. SOF/VEL using the updated drug prices (57). However, for HCV genotype 1, SOF/VEL rather than SOF/LDV was cost effective in this study which was different from our study. The economic difference of SOF/VEL between the two studies was most likely caused by the different SVR rates. SVR rates of SOF/LDV and SOF/VEL in our study were 99.61%, (CI: 99.02%, 1) and 100% (CI: 99.46%, 1) respectively. However, SVR rates of SOF/LDV and SOF/VEL by Chen et al. were 94.9% (CI: 93.3, 96.4%) and 98.5% (CI: 97.1, 99.9%) respectively, where SOF/VEL was statistically better than SOF/LDV in terms of clinical effect. Another possible reason for the different conclusions can be attributed to the definition of the target population. Our cohort was homogenous consisting of treatment-naïve pre-fibrosis patients which was different from the heterogenous cohort of treatment experience and fibrosis stages modeled by Chen et al. (57).

SOF/LDV and SOF/VEL have been assessed economically in various decision-making settings previously (15–17). Without exception, price was a big obstacle to realize their value in comparison with IFN-based regimens. The study by Chen et al. proposed an 81% price cut for the 12-week SOF/LDV course in mainland China. Our results confirmed this proposal by finding that, with an 85% price cut through government-industry negotiation, SOF/LDV met the cost-effectiveness standard. Complimentary to another study where the SOF/LDV regimen showed cost-effectiveness in treatment-experienced patients (15), the present study suggested that SOF/LDV was also cost-effective in treatment-naïve patients.

Prices of the three drugs were among the 5 most influential factors in pairwise DSA analyses (Figures 2, 3). In the DSA comparing SOF/VEL with SOF/LDV, the economic value of both drugs was greatly affected by their prices. If the price of SOF/VEL was reduced by 40%, which is highly likely under the current Chinese policy and government commitment (48), SOF/VEL would be the optimal strategy compared to SOF/LDV. The cost-effective choice between SOF/VEL and SOF/LDV appears to be based on price, which was also the case for other DAAs. Given the excellent clinical profile of the DAA regimens, price-cutting has become the major strategy to make gains in the market. Presently there is a trend where DAA prices are driven down due to competition from generic products, pressure from media and patient groups, licensing agreements between pharmaceutical companies and governments in low-middle income countries. The large initial medical expenditure that was criticized in the early DAA era does not appear to be an issue at present (49, 50).

Universal coverage is the key to the success of the WHO global HCV elimination target (6) and the DAA price has been a major obstacle (51). Partly as a response to the WHO target, China implemented a policy called the New Cities Centralized Drug Procurement in 2018 where the price of brand drugs was dramatically cut to improve drug affordability and accessibility (10). Both SOF/VEL and SOF/LDV to date have had their prices cut by 85%, and hence were qualified for reimbursement (11). As a result, two SOF-based regimens were found cost-saving compared to the traditional regimen in our study. In the future more DAAs are expected to enter the NRDL and further the volume-based procurement plan. Price-cutting coupled with reimbursement would improve the universal coverage of DAA. China has taken a step closer to the WHO target (6), although it is not fully on track yet (52). Our findings indicate an optimistic future.

Treatment duration of SOF/VEL, pegIFN, RBV and SOF/LDV were important in determining the relative cost-effectiveness in the DSA. Shortening the DAA duration will intuitively reduce the upfront cost and likely add to the long-term economic value of DAA. The course of several DAA regimens has already been shortened from 24 weeks to 12 weeks (53). Shorter treatment duration is also associated with high compliance and drug persistence. However, whether shortening the treatment duration improves cost-effectiveness is still at debate (54, 55). To date clinical evidence is mixed with regard to SVR obtained through a reduced treatment duration (56). This is a promising area for future research.

Although SOF/VEL was not as cost-effective as SOF/LDV, SOF/VEL like SOF/LDV was statistically dominant to pegIFN + RBV despite the nearly identical SVR rates between them. This similar pattern, where the DAAs were economically superior to pegIFN + RBV despite their differences, also emerged when comparing other DAAs. Since most IFN-free regimens have been found to be either cost-saving or cost-effective compared to pegIFN + RBV (18, 19, 57, 58), IFN-based regimens may soon need to be replaced by IFN-free regimens. In addition, the price of pegIFN is relatively higher in itself. A comprehensive assessment of the value of pegIFN is warranted. Recent studies found that, when combined with DAAs, pegIFN could shorten treatment duration with minimal or no impact on the SVR (59). Combination regimens also achieved high SVR rates in difficult-to-cure patients for whom pegIFN-free regimens were not as effective. Therefore, the future use of pegIFN is to optimize or personalize DAA regimens.

Age of starting drug treatment appeared as the most influential factor in determining comparative cost-effectiveness and net benefit of each regimen (Figures 2, 3, Supplementary Table 1). The younger a patient started DAA treatment, the more QALYs he/she would obtain from DAA regimens compared to pegIFN + RBV. However, studies have shown that the average age of receiving DAA treatment in China is about 45 years (4, 44), which is much older than the age suitable for DAA treatment (60). To date Chinese studies have explored the use of DAA in adolescents (61). Curing young CHC patients can not only improve overall wellbeing, but also prevent future transmission. The limits to prescribe DAAs to specific patients should be relaxed or lifted (12).

One advantage of our study was that we used more Chinese-specific data to inform decision making in a Chinese setting (16, 18, 19, 62). The up-to-date SVR data of SOF/VEL, SOF/LDV and pegIFN + RBV were extracted from recent studies on Chinese patients. The probabilities of receiving a LT and LT-related death are specific to the local healthcare system which determines the availability of donated livers and the quality of usual care. Therefore, we used data from the China Liver Transplant Registry (37). The biggest difference in methodology distinguishing our study from others is that we assumed post-SVR fibrosis progression. Previous studies neglected post-SVR fibrosis progression unless patients had already developed cirrhosis (16, 30, 62). This may not be scientifically sound as fibrosis continued to progress irrespective of SVR (40). Not incorporating post-SVR progression from the model would risk overestimating the value of DAAs.

Some limitations were noteworthy in our study. Subgroup analysis was not conducted due to the lack of data. Thus, our findings may lack power to inform decision-making for some subpopulations. However, an extensive DSA of clinical or epidemiological parameters partially illustrated the cost-effectiveness of the three drugs for different subgroups. SVRs were obtained from study samples heterogenous of fibrosis stages and treatment experience, while our target population was a homogenous cohort of treatment-naïve patients prior to fibrosis development. Thus, inputted SVRs underestimated the clinical efficacy of the three strategies, especially SOF/LDV and SOF/VEL. The cost-effectiveness of DAA regimens has been undervalued accordingly. We did not include combination regimens of pegIFN with SOF/LDV or SOF/VEL when such regimens were not uncommon in current clinical practice. However, findings from our study can be safely extended to combination regimens.

## Conclusion

SOF/LDV appears to be the most cost-effective treatment for CHC patients infected with HCV genotype 1 in China. This finding provides economic evidence to assist clinicians in choosing the best SOF-based regimen. SOF/LDV and SOF/VEL at a micro-level have shown their economic dominance to the traditional SoC, indicating that replacing pegIFN + RBV with DAAs could be a promising national strategy to achieve the WHO global HCV elimination goal. Although the price of DAAs is no longer an issue, further price-cutting would be beneficial to patients and society alike.

## Data Availability Statement

The original contributions presented in the study are included in the article/Supplementary Material, further inquiries can be directed to the corresponding author/s.

## Author Contributions

HZ conceptualized, constructed and analyzed the model, and drafted the manuscript. JC collected and complied literature and data. HS carried out project management and methodology development. NN conceptualized the model, provided clinical insights, and interpreted the results. SS interpreted the results and drafted the manuscript. PW designed the study and drafted the manuscript. YF collected and assembled the data. SZ oversighted the research activities and revised the draft critically. All authors revised the manuscript critically and approved the manuscript for publication.

## Conflict of Interest

The authors declare that the research was conducted in the absence of any commercial or financial relationships that could be construed as a potential conflict of interest.

## Publisher's Note

All claims expressed in this article are solely those of the authors and do not necessarily represent those of their affiliated organizations, or those of the publisher, the editors and the reviewers. Any product that may be evaluated in this article, or claim that may be made by its manufacturer, is not guaranteed or endorsed by the publisher.
