# Supplementary material for: Cost-Effectiveness Analysis of Pan-Genotypic Sofosbuvir-Based Regimens for Treatment of Chronic Hepatitis C Genotype 1 Infection in China
Source: Front Public Health. 2021 Dec 9;9:779215. doi: 10.3389/fpubh.2021.779215 (PMC8695807; doi:10.3389/fpubh.2021.779215)
Supplement: Supplementary file 1 [file Table_1.DOCX]

**Supplementary Table: Impact of model parameters on cost-effectiveness of three strategies in terms of net monetary benefit**

| **Parameter** | **Range of parameter** | **Relationship with NMB** | **Range of NMB ($)** | **Optimal strategy^a^** |
| --- | --- | --- | --- | --- |
| **Age starting drug treatment (year)** | (15 - 70) | Negative | (256,386 - 751,224) | SOF/LDV |
| **Discount rate (%)** | (0 - 5) | Negative | (440,805 - 872,240) | SOF/LDV |
| **Annual direct medical cost for F0-3 ($)** | (593 – 7,565) | Negative | (486,811 - 578,369) | SOF/LDV |
| **Utility of patients in F0/F1 with SVR** | (0.806 - 1) | positive | (510,759 - 589,494) | SOF/LDV |
| **Utility of patients in F2 with SVR** | (0.791 - 1) | positive | (546,051 - 570,820) | SOF/LDV |
| **Utility of patients in F3 with SVR** | (0.766 - 1) | positive | (553,153 - 566,271) | SOF/LDV |
| **Cost reduction due to SVR (relative risk)** | (0.592 - 0.855) | Negative | (554,300 - 565,059) | SOF/LDV |
| **Utility of patients in F0/F1 without SVR** | (0.751 - 0.985) | positive | (556,070 - 563,813) | SOF/LDV |
| **Utility of patients in Cirrhosis with SVR** | (0.722 - 0.955) | positive | (556,382 - 563,464) | SOF/LDV |
| **SVR-induced progression reduction from cirrhosis to DCC (relative risk)** | (0.04 - 0.59) | Negative | (557,531 - 561,139) | SOF/LDV |
| **SVR-induced progression reduction from cirrhosis to death (relative risk)** | (0.1 - 0.52) | Negative | (558,778 - 560,977) | SOF/LDV |
| **Death rate of cirrhosis patients (%)** | (1.38 - 4.08) | Negative | (559,653 - 560,902) | SOF/LDV |
| **Annual direct medical cost for HCC ($)** | (4,618 – 97,943) | Negative | (559,564 - 560,439) | SOF/LDV |
| **Price of SOF/LDV (400/90 mg) ($)** | (5.5 - 16.5) | Negative | (559,580 - 560,345) | SOF/LDV |
| **Regression from F34 to F23 (%)** | (8.39 - 20.17) | Negative | (560,070 - 560,702) | SOF/LDV |
| **Treatment duration of SOF/LDV (weeks)** | (8 - 16) | Negative | (559,969 - 560,577) | SOF/LDV |
| **SVR of SOF/LDV regimen (%)** | (99.02 - 100) | positive | (559,936 - 560,495) | SOF/LDV |
| **Transition from F1 to F2 (%)** | (7.4 - 9.1) | Negative | (559,987 - 560,546) | SOF/LDV |
| **Transition from F0 to F1 (%)** | (9.7 - 11.8) | Negative | (560,033 - 560,510) | SOF/LDV |
| **Transition from cirrhosis to DCC (%)** | (3.8 - 5.3) | Negative | (559,992 - 560,403) | SOF/LDV |
| **SVR-induced progression reduction from cirrhosis to HCC (relative risk)** | (0.16 - 0.27) | Negative | (560,052 - 560,458) | SOF/LDV |
| **Transition from cirrhosis to HCC (%)** | (1.7 - 2.1) | Negative | (560,189 - 560,357) | SOF/LDV |
| **Utility of patients in F2/F3 without SVR** | (0.701 - 0.985) | positive | (560,182 - 560,341) | SOF/LDV |
| **Transition from F2 to F3 (%)** | (10.7 - 12.9) | Negative | (560,194 - 560,339) | SOF/LDV |
| **Utility of patients with HCC** | (0.532 - 0.821) | positive | (560,227 - 560,313) | SOF/LDV |
| **Transition from F3 to cirrhosis (%)** | (10.4 - 13.1) | positive | (560,238 - 560,313) | SOF/LDV |
| **SAE of SOF/LDV regimen (%)** | (0.9 - 4.06) | Negative | (560,236 - 560,294) | SOF/LDV |
| **Death rate of DCC patients in 1st year (%)** | (5.2 - 26) | Negative | (560,248 - 560,293) | SOF/LDV |
| **Annual direct medical cost for LT during 1st year ($)** | (36,370 – 76,923) | Negative | (560,245 - 560,288) | SOF/LDV |
| **DCC to LT (%)** | (0.03 - 10.4) | positive | (560,256 - 560,297) | SOF/LDV |
| **Utility of patients in cirrhosis** | (0.67 - 0.907) | positive | (560,252 - 560,292) | SOF/LDV |
| **Utility of LT patients from 2nd year onwards** | (0.636 - 0.85) | positive | (560,250 - 560,285) | SOF/LDV |
| **Transition from DCC to HCC (%)** | (2.1 - 6.8) | Negative | (560,252 - 560,286) | SOF/LDV |
| **Death rate of DCC patients from 2nd year (%)** | (12.09 - 16.52) | Negative | (560,261 - 560,285) | SOF/LDV |
| **Annual direct medical cost for LT 2nd year onwards ($)** | (6062 - 10103) | Negative | (560,263 - 560,284) | SOF/LDV |
| **Probability of HCC patients receiving LT (%)** | (0.05 - 4) | positive | (560,263 - 560,281) | SOF/LDV |
| **Utility of LT patients during 1st year** | (0.563 - 0.8) | positive | (560,269 - 560,277) | SOF/LDV |
| **Death rate of HCC patients (%)** | (34.9 - 57.6) | Negative | (560,272 - 560,273) | SOF/LDV |
| **Utility of patients with DCC** | (0.517 - 0.837) | positive | (560,273 - 560,273) | SOF/LDV |
| **Treatment duration of SOF/LDV (weeks)** | (8 - 16) | positive | (560,273 - 560,273) | SOF/LDV |
| **Treatment duration of RBV (weeks)** | (24 - 72) | positive | (560,273 - 560,273) | SOF/LDV |
| **Treatment duration of pegIFN (weeks)** | (24 - 72) | positive | (560,273 - 560,273) | SOF/LDV |
| **SVR rate of pegIFN + RBV (%)** | (49.48 - 67.29) | positive | (560,273 - 560,273) | SOF/LDV |
| **SVR of SOF/VEL regimen (%)** | (99.46 - 100) | positive | (560,273 - 560,273) | SOF/LDV |
| **SAE of SOF/VEL regimen (%)** | (0.17 - 2.32) | positive | (560,273 - 560,273) | SOF/LDV |
| **SAE of pegIFN + RBV (%)** | (2.37 - 22.26) | positive | (560,273 - 560,273) | SOF/LDV |
| **Price of SOF/VEL (400/100 mg) ($)** | (11 - 33) | positive | (560,273 - 560,273) | SOF/LDV |
| **Price of RBV (100 mg) ($)** | (0.04 - 1.95) | positive | (560,273 - 560,273) | SOF/LDV |
| **Price of pegIFN (50ug) ($)** | (16.56 - 97.33) | positive | (560,273 - 560,273) | SOF/LDV |
| **Post-LT Death rate in 2nd year onward (%)** | (2.2 - 4.9) | positive | (560,273 - 560,273) | SOF/LDV |
| **Post-LT Death rate in 1st year (%)** | (0.03 - 19.4) | positive | (560,273 - 560,273) | SOF/LDV |
| **Dose of pegIFN (ug)** | (10 - 12) | positive | (560,273 - 560,273) | SOF/LDV |
| **Cost reduction due to SVR (Relative risk)** | (0.592 - 0.855) | positive | (560,273 - 560,273) | SOF/LDV |
| **Annual direct medical cost for DCC ($)** | (2,589 – 50,441) | positive | (560,273 - 560,273) | SOF/LDV |
| **Annual direct medical cost for cirrhosis ($)** | (884 – 26,766) | positive | (560,273 - 560,273) | SOF/LDV |

^a^ Strategy with biggest NMB regardless of parameter variation

DCC, decompensated cirrhosis; F0, METAVIR fibrosis score 0; F1, METAVIR fibrosis score 1; F2, METAVIR fibrosis score 2; F3, METAVIR fibrosis score 3; F4, METAVIR fibrosis score 4; GT, genotype; HCC: hepatocellular carcinoma; HCV, hepatitis C virus; LT: liver transplantation; NMB: net monetary benefit; pegIFN: pegylated interferon; RBV: ribavirin; SAE: serious adverse event; SOF/VEL: sofosbuvir/velpatasvir; SOF/LDV: sofosbuvir/ledipasvir; SVR: sustained virologic response
